# A type of neoplasia deadlier than gastric adenocarcinoma? Report of a case of primary gastric squamous cell carcinoma

**DOI:** 10.1186/s12957-019-1657-x

**Published:** 2019-06-29

**Authors:** Michail G. Vailas, Athanasios Syllaios, Natasha Hasemaki, Maria Sotiropoulou, Eustratia Mpaili, Helen Sarlanis, Evangelos Felekouras, Alexandros Papalampros

**Affiliations:** 1 1st Surgical Department, Athens University School of Medicine, “Laiko” General Hospital, Agiou Thoma 17, 11527 Athens, Greece; 2 3rd Surgical Department, Evangelismos General Hospital, Ypsilantou 47, 10676 Athens, Greece; 3 Pathology Department, Athens University School of Medicine, “Laiko” General Hospital, Agiou Thoma 17, 11527 Athens, Greece

**Keywords:** Carcinoma, Gastric, Metastases, Primary, Squamous, Stomach

## Abstract

**Background:**

Primary gastric squamous cell carcinoma is an extremely rare malignancy with few case reports reported so far in the current medical literature. Its incidence varies between 0.04 and 0.07% of all gastric malignancies with a male predominance in the sixth decade of life. It has been found that this type of malignancy has a more aggressive behavior and associated poorer prognosis, when compared to gastric adenocarcinoma. Thus, the most appropriate management of this kind of neoplasia is still debatable due to the small number of reported cases.

**Case presentation:**

We report the case of a 66-year-old man who underwent total gastrectomy with D2 lymphadenectomy for an ulcerative lesion in the fundus of the stomach that turned out to be primary gastric squamous cell carcinoma.

**Conclusions:**

Upon confirmation of this specific malignancy, the affected patients should be enrolled in strict follow-up protocols after curative surgery, since the risk for metastasis is high. Physicians should maintain high clinical suspicion in order to diagnose these tumors at an early stage, along with the need to rule out any other possible primary sites of squamous malignancy.

## Background

Primary gastric squamous cell carcinoma (SCC) is an extremely rare entity with less than 100 cases described in the current medical literature. It represents 0.04–0.07% of all gastric cancers with an incidence ratio of men to women of about 5:1. The most common tumor location is the upper third of the stomach, and the prevalence is higher in the sixth decade of life. However, a wide spectrum of ages affected has been reported [1]. The exact pathogenesis of primary gastric SCC is still unknown, but it seems to be more aggressive and prone to lymphovascular invasion compared to adenocarcinoma. The prognosis and the management of these uncommon tumors still remain unknown due to the low incidence of gastric SCCs. We describe a case of a 66-year-old man who was diagnosed with primary SCC of the fundus of the stomach. He underwent curative resection with total gastrectomy and D2 lymphadenectomy, but, due to the advanced stage of his disease, 2 months after the operation metastases to the lungs were apparent and adjuvant chemotherapy was initiated.

## Case presentation

A 66-year-old man was referred to our surgical outpatient clinic because of an endoscopic report that was indicative of a small irregular ulcerative lesion in the fundus of the stomach. The histopathological report was consistent with gastric squamous carcinoma (AE1/AE3+, p40+, Chromogranin A−, Synaptophysin−, c-erB-2/HER2−). The patient complained of epigastric pain and abdominal cramps for the last 2 months with no incident of hematemesis, melena, or hematochezia. He had an unintentional weight loss of approximately 10 kg during the last 4 months. He was suffering from type 2 diabetes with a past medical history of smoking, 100 packs per year approximately. The patient had never undergone a surgical operation before.

**Fig. 1:**
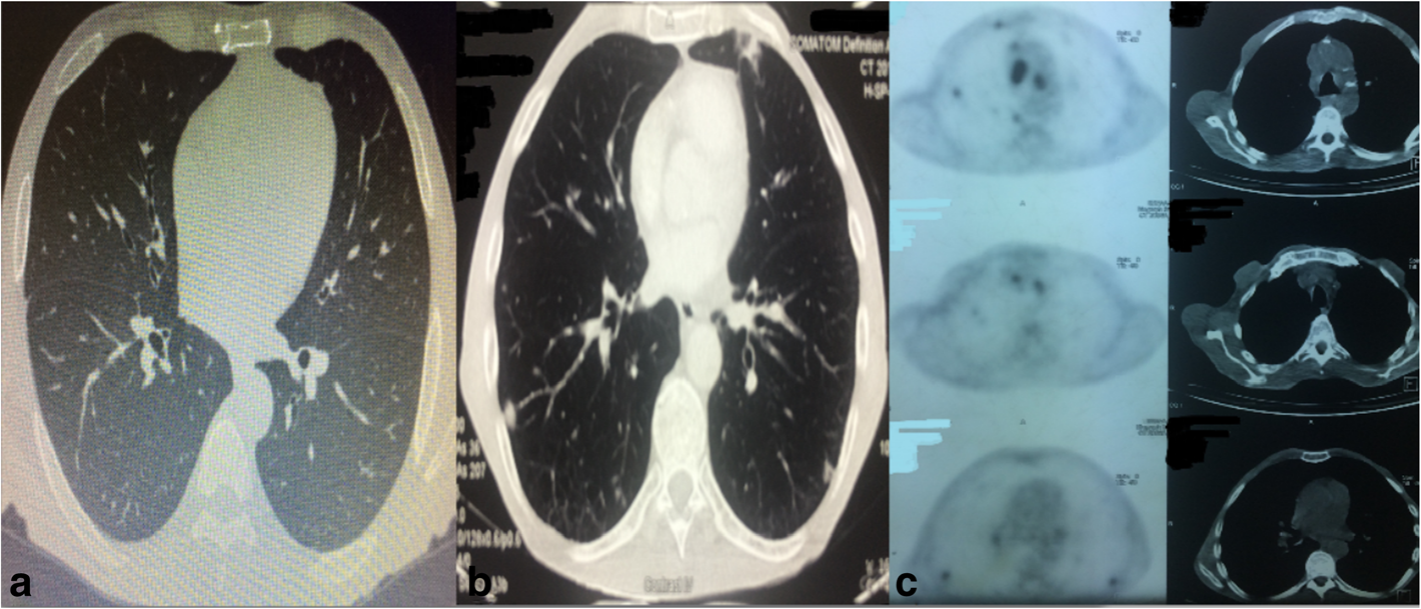
**a** Preoperative CT thorax showing the absence of metastasis. **b** Postoperative CT thorax with metastatic lung lesions. **c** PET-CT confirming the diagnosis of metastatic lung lesions

His blood tests showed chronic anemia and his tumor markers (CEA, CA 19-9, aFP, and PSA) were within normal limits. The abdominal computed tomography (CT) revealed no other sites of tumor location or other primary sites of squamous malignancy (Fig. 1). Maximum diameter of the tumor was 3 cm in the fundus of the stomach with no suspicious lymph nodes, while the chest CT was normal. The multidisciplinary team decided to proceed to surgery. Three weeks after the multidisciplinary team meeting due to the high volume of the patients, the patient underwent total gastrectomy with Roux-en-Y reconstruction, along with splenectomy and D2 lymphadenectomy. The postoperative recovery was uneventful and the patient was discharged on postoperative day 12.

**Fig. 2:**
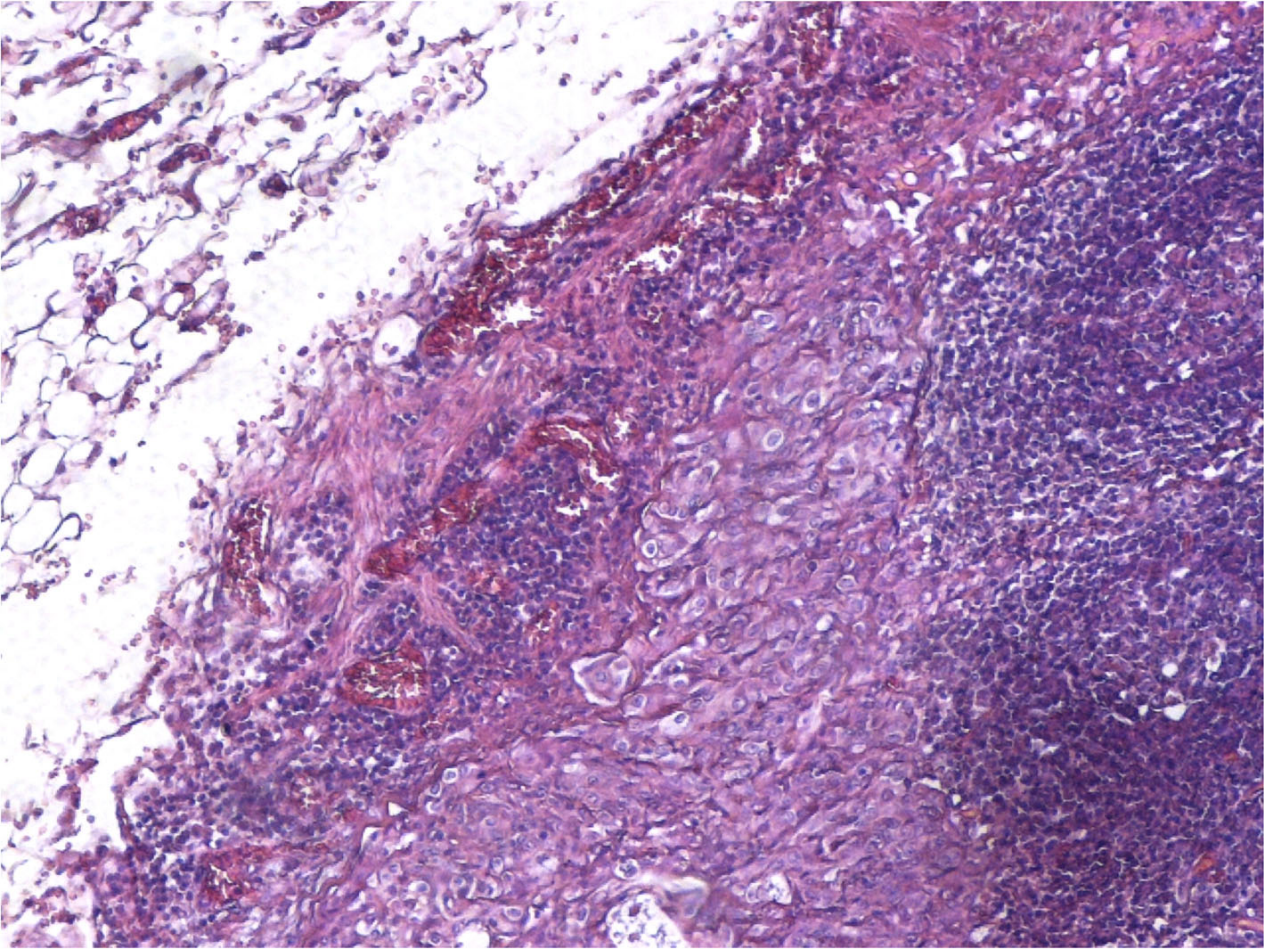
Poorly differentiated clear cell gastric squamous cell carcinoma

**Fig. 3:**
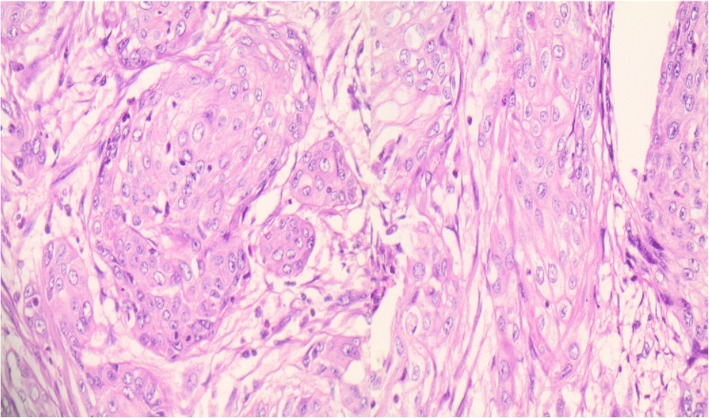
Poorly differentiated clear cell gastric squamous cell carcinoma with subtle intracellular bridging (H/E stain × 40)

**Fig. 4:**
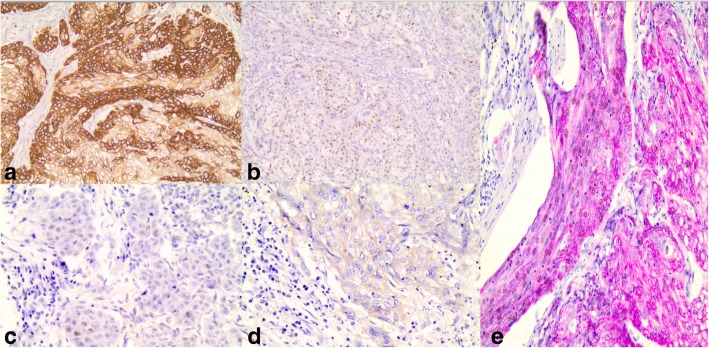
Immunohistochemistry of gastric tumor confirming the diagnosis: **a** positive CK14 stain (× 20), **b** positive cyclin-D1 stain (× 20), **c** positive p53 stain (× 40), **d** positive EGFR stain (× 40), and **e** co-expression of CK5/6 and p63 stains (× 20)

Pathological assessment of the specimen revealed a 4.7 × 3 × 2.5-cm mass located in the fundus of the stomach invading the subserosa. Out of 48 lymph nodes resected, 9 of them were positive for tumor infiltration. Perineural invasion and extramural venous invasion were also found. The resection margins were free and the final TNM stage was T3N3aM0 (WHO 2017). Microscopically, the tumor was a poorly differentiated gastric squamous cell carcinoma with clear cell characteristics, chronic inflammatory, and desmoplastic stromal reaction (Fig. 2). Immunohistochemistry of gastric tumor confirmed the diagnosis with positive CK14 stain, cyclin-D1 stain, p53 stain, EGFR stain, co-expression of CK5/6, and p63 stains (Figs. 3 and 4). In order to rule out other possible diagnoses or other occult primary tumors, extensive immunohistochemistry check including MUC1, uroplakin III, PAX8, RCC, and PAS was performed without positivity.

Because of the rarity of this specific type of tumor in the stomach, the tumor board decided to rule out other possible primary sites of squamous malignancy. The patient underwent a cranial, abdomen, and chest CT, which revealed enlarged lymph nodes in the mediastinum, in right and left paratracheal area, as well as 4 small bilateral possible secondary lung lesions (Fig. 1). Interestingly enough, these findings were not apparent in the preoperative CT scan. However, they were evident 3 weeks after surgery, while preoperative chest CT scan was unremarkable, a fact that underlines the aggressive behavior of this malignancy. Following that, the patient also underwent bronchoscopy which did not reveal any lesion. Endobronchial brushing results were normal. Full-body FDG PET/CT scan showed multiple metastatic chest lesions of maximum diameter 11 mm (Fig. 1). The patient received adjuvant chemotherapy with paclitaxel and carboplatin. To date, 12 months after the initiation of the chemotherapy, there is no progression of the disease, with minor regression of the metastatic chest lesions.

## Discussion

Primary gastric squamous cell carcinoma (PGSCC) is an extremely rare entity with only a few case reports published so far. Adenocarcinoma accounts for approximately 95% of all gastric malignancies, while PGSCC incidence is only 0.04–0.07%. As far as gender predominance is concerned, male to female ratio is reported to be approximately 4–6/1 [2, 3]. Its prevalence is higher in the sixth decade of life, and the most common tumor location like in our patient seems to be the upper third of the stomach (57.1%), followed by the lower third (21.4%) and the middle third (19.6%) [1, 2]. As in our patient, most of the patients have a long history of smoking, a fact that has been implicated to promote the occurrence of this type of tumor [3]. In this study, 13 patients (61.9%) had a long history of smoking, and according to our observation, like lung squamous carcinoma and esophageal squamous carcinoma, a long history of smoking may promote the occurrence of this tumor.

Diagnostic criteria that were first described in 1967 by Parks were the following: (a) the tumor should not be located at the cardia, (b) the tumor should not extend into the esophagus, and (c) there should be no evidence of SCC in any other parts of the body [4]. All of the above criteria had to be met, but the Japanese Gastric Cancer Association later in 2011 suggested new criteria including the following: (a) all tumor cells have to be SCC cells without any gland cancer cells and (b) SCC must originate in the gastric mucosa [5]. Our patient met the updated criteria for making the diagnosis of PGSCC, as all tumor cells were SCC cells and the lung lesions were metastases from the primary gastric tumor. As there are currently no clinical characteristics that distinguish patients with gastric adenocarcinoma from patients with SCC, histopathological examination is required to confirm the diagnosis [6]. Individual cell keratinization, keratin pearls, intercellular bridges, and positive immunoreactivity for p63 and CK5/6 may be found. Blood examinations, biochemical tests, and tumor serum markers may reveal anemia (66.7%), hypoalbuminemia (42.9%), hypocalcemia (42.9%), elevated values of CEA (38%), and CA19-9 (33.3%) [3].

Clinical characteristics are not pathognomonic as they are the same with nearly every symptomatic gastric malignant tumor: nonspecific abdominal pain, nausea, vomiting, weight loss, vomiting, melena, bloating, and early satiety [5]. The exact origin and pathogenesis of the tumor is not well known, but several mechanisms have been proposed such as the presence of totipotential (stem) cells, the existence of areas of ectopic squamous cell nests, squamous differentiation of preexisting adenocarcinoma, squamous metaplasia of glandular epithelium secondary to chronic mucosal damage, and SCC arising from the vascular endothelium of the stomach. Furthermore, Epstein-Barr virus infection has been lately implicated in the pathophysiology of the disease [3, 7].

Currently, there is no consensus on how to treat this disease, because of the fact that primary SCC of the stomach is rare and the current evidence is based on case reports and small case series. Radical surgical excision with lymph node dissection remains the main therapeutic approach and has been proposed as the only potential cure for localized disease as it can improve the prognosis of gastric SCC [5, 8, 9]. The efficacy of systemic chemotherapy against the recurrence or metastasis of PGSCC has been demonstrated in some studies [2, 10]. Adjuvant chemotherapy consisting of 5-fluorouracil-based regimens; platin- and taxane-based regimens such as docetaxel + oxaliplatin/cisplatin + fluorouracil, flouorouracil + oxaliplatin + calcium folinate (FOLFOX), capecitabine + oxaliplatin (XELOX); and other combinations has been used effectively in the treatment of PGSCC, offering better outcomes regarding survival, recurrence, and prognosis [5, 6]. Neo-adjuvant chemotherapy for PGSCC seems to be beneficial and efficient but the currently available data remains limited [3, 5, 11].

The prognosis of PGSSC when compared to gastric adenocarcinoma seems to be worse, as it is usually diagnosed at an advanced stage, metastasizing in the liver, lymph nodes, and other organs [3, 12]. Meng et al. reported that median survival of patients with recurrent or metastatic gastric SCC is about 7 months, whereas patients with advanced adenocarcinoma of the stomach show a median survival of 11 months [6].

Even though clinical features and epidemiological characteristics of PGSCC have been reported in the literature, to date, no standard treatment strategy has been defined. The adoption of current therapeutic strategies of gastric ADC in the management of gastric SCC is debatable, due to differences in molecular characteristics, tissues of origin, and prognosis. The dilemma is whether to manage PGSCC according to therapeutic principles of gastric ADC or that of esophageal SCC. To date, the treatment of PGSCC tends to follow that of gastric ADC; however, a standard chemotherapy regimen for PGSCC has not yet been established. Independently of the optimal chemotherapeutic agent, R0 resection remains the mainstay of the treatment. Multimodality treatments have been applied to improve the overall outcomes of esophageal SCC, especially for patients with locally advanced tumors. Based on several meta-analyses and randomized controlled trials, neoadjuvant chemoradiotherapy, neoadjuvant chemotherapy, and definitive chemoradiotherapy are considered acceptable treatment modalities for locally advanced esophageal SCC in guidelines from the European Society for Medical Oncology (ESMO). However, not much information is available on the role of neoadjuvant chemoradiotherapy in gastric SCC. The optimal multimodality regimen has yet to be defined.

Preoperative radiotherapy (RT) was envisaged to increase the possibility of negative circumferential margins, to lower the loco-regional recurrences and to improve survival. However, there is no randomized control trial (RCT) comparing preoperative RT followed by surgery to surgery alone for PGSCC. Concerning the treatment of esophageal SCC, researchers concluded that there is not enough evidence to suggest that neoadjuvant RT improves the survival of patients with esophageal SCC, implicating that the role of neoadjuvant RT in the management of PGSCC also remains uncertain. To detect reliably a potential benefit of neoadjuvant RT, trials or a meta-analysis would be needed.

Literature search retrieved only a few similar reported cases of PGSCC, with the majority of them describing one single case (Table 1). Numerous previous studies have provided conflicting evidence regarding the optimal therapeutic approach and prognosis of PGSCC. The reported studies indicate that radical surgical excision can improve the prognosis of PGSCC and is the only potential cure for localized disease. Although the effects of chemotherapy on advanced gastric SCC have previously been described in case reports, only a few studies have demonstrated the efficacy of systemic chemotherapy against the recurrence or metastasis of primary SCC of the stomach. In the present study, similar to the cases reported in the literature, we report a case of a 66-year-old male with the preoperative diagnosis of gastric SCC. Radiological imaging and endoscopy were indicative of a T2N0M0 gastric cancer; hence, the multidisciplinary team decided to proceed to surgery, without administration of neoadjuvant treatment. Nonetheless, in our department, we do not routinely perform diagnostic laparoscopy. We performed a total gastrectomy with Roux-en-Y reconstruction, along with splenectomy and D2 lymphadenectomy, followed by adjuvant chemotherapy with paclitaxel and carboplatin.

**Table 1:** Summary of case reports with primary gastric squamous cell carcinomas

Author	Year	Age (years)	Sex	Position	Survival (months)
Raju et al. [13]	1987	59	M	ps	n/a
Schmidt et al. [8]	2001	61	M	ps	70
Dursun et al. [12]	2003	65	M	Lc	3
Hara et al. [14]	2004	85	M	Gc	17
Choi et al. [15]	2007	40	M	Gc	12
Callacondo et al. [16]	2009	83	M	Antrum	24
Guttmann et al. [17]	2012	81	F	Lc	n/a
Tokuhara et al. [18]	2012	67	M	Lc	13
Little et al. [19]	2013	73	M	Antrum	n/a
Hwang et al. [20]	2014	61	M	Fundus	6
Wakabayashi et al. [2]	2014	69	M	Lc	36
Shi et al. [21]	2014	66	M	Fundus	n/a
Mardi et al. [22]	2015	42	M	Antrum	n/a
Gao et al. [23]	2015	50	M	Antrum	3
Modi et al. [24]	2015	55	M	Lc	n/a
Wu et al. [25]	2016	59	M	EGJ	16
Segura et al. [26]	2016	64	F	Fundus	n/a
Gülçiçek et al. [27]	2016	49	M	Antrum	n/a

## Conclusion

PGSCC is an extremely rare malignancy of the stomach and seems to be a highly aggressive tumor with poor prognosis. More data and larger series are needed in order to safely decide the best treatment option for these patients and whether adjuvant along with neo-adjuvant treatment plays a significant role in the management of these tumors. Physicians should be aware of its existence, and a high clinical suspicion is required to rule out other possible primary or secondary sites of this specific type of malignancy. An intensive follow-up is imperative after curative surgery, due to the highly aggressive behavior of these tumors.

## Data Availability

Not applicable

## Abbreviations

PGSCC: Primary gastric squamous cell carcinoma
SCC: Squamous cell carcinoma
